# In Vitro Antibacterial and Antioxidant Activities and Molecular Docking Analysis of Phytochemicals from *Cadia purpurea* Roots

**DOI:** 10.1155/2022/4190166

**Published:** 2022-02-24

**Authors:** Tsegu Kiros, Rajalakshmanan Eswaramoorthy, Yadessa Melaku, Aman Dekebo

**Affiliations:** ^1^Department of Applied Chemistry, School of Applied Natural Sciences, Adama Science and Technology University, P.O. Box 1888, Adama, Ethiopia; ^2^Central Laboratory, Haramaya University, P.O. Box 138, Dire Dawa, Ethiopia; ^3^Department of Biomaterials, Saveetha Dental College and Hospitals, Saveetha Institute of Medical and Technical Sciences (SIMATS), Chennai, India

## Abstract

Phytochemicals and antibacterial and antioxidant activities of *Cadia purpurea* roots were investigated herein for the first time. The phytochemical study led to the isolation of two compounds, di-(2-methylheptyl) phthalate (**1**) and 13-O-pyrrolecarboxyl lupanine (**2**), from methanol roots extract of *C. purpurea*. The antibacterial activity results revealed that the *n*-hexane extract presented a better inhibitory value (13.8 ± 0.0 mm) followed by chloroform (11.1 ± 0.4 mm) and chloroform : methanol (1 : 1) (10.7 ± 0.1 mm) extracts against *E. coli* at the maximum dose of 100 mg/mL. While, methanolic and ethanolic extracts displayed a mild activity against same bacterium at same dose. The methicillin resistant *S. aureus* was found with almost total resistance to all extracts up to the 100 mg/mL. The chloroform : methanol (1 : 1), chloroform, and *n*-hexane extracts recorded inhibition zone values (8.0 ± 0.0–10.0 ± 0.1 mm, 7.7 ± 0.0–9.8 ± 0.1 mm, and 7.3 ± 0.2–8.9 ± 0.2 mm, respectively) better than chloramphenicol (7.2 ± 0.6 mm at 30 *μ*g dose) against *P. aeruginosa*. The alcoholic extracts also exhibited an activity better than chloramphenicol up to 25 mg/mL against same bacterium. Compound **2** produced a comparable inhibition value (9.6 ± 0.0 mm to 18.5 ± 0.0 mm) to that of chloramphenicol (21.5 ± 0.3 mm) against *E. coli* at doses up to 1.0 mg/mL; whereas, compound **1** showed a slight activity (7.1 ± 0.1 mm–10.3 ± 0.0 mm). Both compounds were found generally inactive against *S. aureus*, while they provided an activity better than chloramphenicol (7.2 ± 0.6 mm) against *P. aeruginosa* with inhibition zones ranging from 7.1 ± 0.0 mm to 9.0 ± 0.1 mm for compound **1** and 7.2 ± 0.0 mm to 10.6 ± 0.0 mm for compound **2**. Ethanolic and methanolic extracts exhibited a better DPPH radical scavenging activity (IC_50_ values of 12.9 and 16.03 *μ*g/mL, respectively) and strong ferric ion reducing power (with absorbance of 0.788 ± 0.000 and 0.810 ± 0.001, respectively) at a concentration of 500 *μ*g/mL compared to the other extracts. Compound **1** also possessed a better anti-DPPH trapping activity (IC_50_, 7.99 *μ*g/mL) than compound **2** (IC_50_, 58.34 *μ*g/mL). The compounds, however, indicated a weak ferric ion reduction power even at higher amount. In general, the observed antibacterial and antioxidant activities of isolated compounds and extracts were found to be dose-dependent. Conducting further biochemical investigations on all parts of this plant could provide opportunities of finding extra alkaloidal compounds and other phthalate derivatives with better biological activity.

## 1. Introduction

*Cadia purpurea* (Fabaceae) is a shrub which inhabits rift valley escarpment and bush land, commonly in altitudes about 1300–2700 m. In Ethiopia. it is found in Tigray, Welo, Shewa, Hararge, and Bale regions. *C. purpurea* is also distributed in Eritrea, Yemen, Oman, North Somalia, and North Kenya [1]. In Ethiopia, the root of this plant is widely used traditionally to treat severe wounds [2] and the leaves are applied for the treatment of fire burn by mixing the powder with coffee in the Northern part of Ethiopia [3]. Also, the leaves are used for the treatment of wound infection and nail inflammation in Eritrea [4]. The nectar of the plant is also applied for reducing gastritis, heart burn, and pyrosis by communities of Harla and Dengego valleys, Eastern Hararge, Ethiopia [5]. Previous reports showed the presence of flavonoids and alkaloids as principal constituents of different parts of *C. purpurea*. Flavonoids such as apigenin, apigenin 7-O-glucoside, and chrysoeriol were isolated from ethanol leaves extract [6]. Besides, the leaves of *C. purpurea* from Ethiopia yielded new quinolizidine alkaloids, namely, alkaloid I, II, and III [7]. l-Sparteine, lupanine, *α*-amyrin, and cadiamine were also isolated from leaves and twigs of the plant of same country, according to Van Rijk and Radema [8]. A novel and naturally occurring derivative of lupanine known as 13-ethoxylupanine was obtained from the ethanol and chloroform extracts of *C. purpurea* [9]. The leaves of *C. purpurea* were also studied for their luteolin content [10].

Despite the traditional use of this plant against wide array of diseases, there was no report on phytochemicals and biological activities of the roots of *C. purpurea* of Ethiopian origin. Therefore, in this study, we present the isolation, antibacterial, and antioxidant activities of the roots' extracts of *C. purpurea*. In the course of the study, the drug-likeness characteristics of isolated compounds was also assessed by docking against *E. coli* DNA gyraseB enzyme (PDB ID: 6F86). In the present study, two compounds, di-(2-methylheptyl) phthalate (**1**) and 13-O-pyrrolecarboxyl lupanine (**2**), were isolated and reported from methanol roots extract of *C. purpurea*. This finding also indicated that isolated compounds and crude extracts of the studied plant showed a dose-dependent antibacterial and antioxidant activities. The result of molecular docking analysis revealed that compound **1** shows two hydrophobic interactions with Ile-78 and Ile-94, a *π*-anion interaction with Glu-50 and other Van der Walls interaction with some specific active site pockets of 6F86. Whereas, compound **2** makes three H-bond interactions with Thr-165, Gly-77, and Ile-78 and a Van der Walls interaction with Asp-73 residual amino acids. Besides, the two compounds form a coordination with Pro-79 active site. In comparison to ciprofloxacin, the drug-likeness property of compound **1** is expressed in terms of a hydrophobic interaction with Ile-78 and a *π*-anion interaction with Glu-50 binding sites of the protein model. Whereas, compound **2** looks like ciprofloxacin via the formation of a H-bond with Thr-165 amino acid residue. Therefore, further biochemical investigations on *Cadia purpurea* may lead to the finding of potential phytochemicals with better biological activities and drug-likeness characteristics.

## 2. Materials and Methods

### 2.1. Plant Material

**Figure 1:**
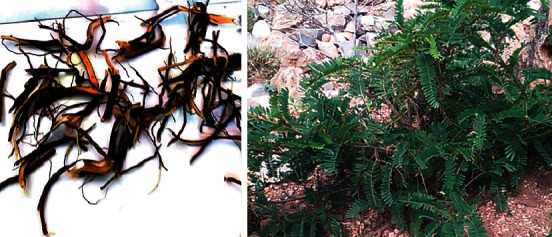
*Cadia purpurea* shrub and its root part (photo by Tsegu K., September 2019).

Roots of *Cadia purpurea*Figure 1 were collected from Harla and Dengego mountains during September 2019. Harla and Dengego mountains are found under Dire Dawa administrative town in Eastern Ethiopia situated at 515 km east of Addis Ababa. They are delimited with coordinates of 9º27′ and 9º39′N latitude and 41º38′ and 42º20′E longitude with elevation ranges between 950 and 2260 m above the sea level. The mean annual temperature is about 22.8°C, ranging from a mean minimum of 16.2°C to mean maximum of 30.4°C. The mean annual rainfall in the surrounding areas ranges from about 1,000 to 600 mm [5]. The botanical name of the collected plant species was confirmed using the flora data of Haramaya University, Ethiopia, via visual comparison with the already authenticated plant specimens preserved at the herbarium of the university. A voucher specimen was deposited in the herbarium of Haramaya University (accession number, AHU178) with the help of Dr. Anteneh belayneh. Collected roots were air dried in shade at room temperature at central laboratory of Haramaya University and ground using an electrical commercial laboratory blender. Powdered material was kept in an airtight glass container at 4°C refrigerator until the extraction process was commenced.

### 2.2. Chemicals, Apparatuses, and Instruments

The chemicals, reagents, apparatuses, and instruments used in the present work were as follows: TLC visualizing reagents (iodine vapor and vanillin/H_2_SO_4_); analytical grade chemicals (methanol, ethanol, *n*-hexane, chloroform, dichloromethane, ethyl acetate, acetone, acetic acid, ascorbic acid, DPPH radical, ferric chloride, potassium ferricyanide, 0.2 M potassium phosphate buffer, trichloroacetic acid, DMSO, chloramphenicol, anhydrous Na_2_SO_4_, MHA, and silica gel, 60–120 mesh size); and apparatuses and instruments such as precoated aluminum TLC sheet silica gel 60 F_254_ (Merck), TLC chamber, capillary tube, PTLC, glass column, waring commercial laboratory blender (Torrington, CT., USA), shaker (Hy-5A, Movel Scientific Instrument Co., Ltd., China), rotary evaporator (rotary vacuum, Jainsons, India), suction filtration apparatus, Petri plates, incubator (Binder B28, Germany), autoclave (Tuttnauer 3150EL, Israel), UV-lamp (UVP Chromato-Vue C-70G, Analytik Jena, USA), digital melting point apparatus (SMP10, Bibby Scientific, UK), UV-vis spectrophotometer (Cecil CE4001 UV/VIS, Cambridge, England), Spectrum 65 FTIR, EI GC-MS spectrometry, and ^1^H (400 MHz) and ^13^C (100 MHz) NMR spectroscopy.

### 2.3. Preparation of Crude Extracts

Air-dried roots powder (100 g) of *C. purpurea* was successively extracted with *n*-hexane (1 L, 3x), chloroform, chloroform : methanol (1 : 1), methanol, and ethanol via shaking over an orbital shaker for 24 h, filtered by a suction filtration apparatus, concentrated using a rotary evaporator at reduced pressure to furnish extract yields of 0.60, 0.57, 4.67, 4.78, and 0.20% (w/w), respectively. Obtained extracts were subjected to in vitro antibacterial and antioxidant activity assay and TLC guided column chromatographic fractionation.

### 2.4. Fractionation of Methanol Extract

Methanol extract (3.10 g) was adsorbed on silica gel (20.00 g, 230–400 mesh size) and subjected to silica gel column chromatography. Elution was carried out using EtOAc/MeOH with increasing polarity, and eighty (80) fractions were collected. The TLC profile of collected fractions were examined using EtOAc/MeOH/AcOH (3 : 1:0.1) and EtOAc/MeOH/AcOH (1 : 1:0.1) as developing solvents and visualized via a UV-lamp (at 254 and 360 nm) followed by iodine vapor. By looking at their TLC chromatogram, fractions with similar spot were combined, concentrated, and their amount was determined. Fractions Fr11-18 (eluted with 100% EtOAc, 45 mg) and Fr72-88 (eluted with 1 : 1 ratio of EtOAc/MeOH, 300 mg), which showed good TLC profile, were combined and yielded compounds **1** and **2**, respectively.

### 2.5. Instrumental Conditions

All NMR (^1^H, ^13^C, and DEPT-135) experiments were performed on a Bruker ACQ 400 Avance spectrometer operating at 400 MHz (for ^1^H) and 100 MHz (for ^13^C and DEPT-135) equipped with a 5 mm proton probe and running Topspin 2.1 software at 298K. All chemical shifts (*δ*_ppm_) of the spectra were recorded relative to an internal TMS reference. Obtained spectral data after acquisition were further processed using MestReNova software (Mestrelab Research S.L., Version 12). The FTIR experimental process was run on Spectrum 65 FTIR (PerkinElmer) in the wavenumbers range of 4000–400 cm^−1^ with 4 cm^−1^ resolution and 4 number of scans using the KBr pellets. The measured ASCII data were then converted and processed via OriginPro2019b.Win software into FTIR spectral graphs (% transmittance versus wavenumbers in cm^−1^). In the GC-MS instrumentation, GC analyses were done on 7890B (Agilent Technologies, USA) equipped with an HP 5MS nonpolar column (internal diameter of 30 m × 250 *μ*m and 0.25 *μ*m of film thickness). Helium was used as carrier gas with a flow rate of 1 mL/min. The injector temperature was set at 230°C, and the injection mode was a split mode with 10 : 1 of split ratio. The oven temperature was initially set at 40°C for 5 min and was elevated to 250°C with a rate of 6°C/min held for 20 min. The total run-time was 60 min. The MS part was run on 5977A Network (Agilent Technologies) and operated in the EI mode at 70 eV. All spectra were scanned from 50 to 650 m/z range, and compounds were identified by comparing their mass spectra with those possible compounds searched from the database of NIST11 (National Institute of Standards and Technology, Gaithersburg, USA). Relative amounts of identified compounds were calculated based on the peak areas of the total ion chromatograms (TIC).

### 2.6. In Vitro Antibacterial Activity of Extracts and Isolated Compounds

The antibacterial activity of each extract (*n*-hexane, chloroform, 1 : 1 of chloroform : methanol, methanol, and ethanol) and isolated compounds **1** and **2** from the plant was evaluated against three standard human pathogens, namely, *Staphylococcus aureus* (*S. aureus,* ATCC 25923), *Escherichia coli* (*E. coli,* ATCC 25922), and *Pseudomonas aeruginosa* (*P. aeruginosa,* ATCC 27853) bacterial strains which were obtained from Ethiopian Public Health Institute (EPHI). Experimental activity was conducted at microbiology laboratory of Medical Laboratory Department, Haramaya University, in collaboration with microbiologists. The agar medium disc-diffusion technique was followed to evaluate the antibacterial effectiveness of each extract and compound isolate using the standard protocols of Clinical and Laboratory Standards Institute (CLSI) [11]. Briefly, few colonies (3–5) of similar morphology of bacterial strains were transferred with a sterile inoculating loop aseptically into a liquid medium (saline solution). The turbidity was adjusted equivalence to 0.5 McFarland standard solution (10^8^ CFU/mL) using the McFarland background. Bacterial suspension was then inoculated by swapping with cotton swap onto Petri plates containing Mueller Hinton agar (Hi media) medium prepared as per the manufacturer's instruction. Sterilized Whatman No. 1 filter paper discs (6 mm in diameter) were prepared using a puncher to hold samples. A stock solution of each extract (200 mg in 2 mL) and isolated compound (5 mg in 5 mL) was prepared using 4% DMSO. Three concentrations of 50 mg/mL, 25 mg/mL, and 12.5 mg/mL were prepared from stock solutions of each extract using the two-fold serial dilution method [12]. Four various solutions, 0.5, 0.3, 0.1, and 0.05 mg/mL, of each compound were also prepared from their corresponding stock solutions. Chloramphenicol impregnated standard disc (30 *μ*g) and DMSO solvent were used as standard antibiotic and negative control, respectively. Then, each sample (100 *μ*L), including the positive and negative controls, was loaded onto separate paper discs (as thick as the chloramphenicol disc) followed by putting the discs onto the Petri plates containing the bacterial culture inoculated MHA and incubated them at 37°C for 18–24 h. Inhibition zones were indicated by clear area around the paper disc which were measured by caliper (in mm) to evaluate the degree of susceptibility of the bacterial strains to the tested analytes. Each experiment was done in duplicate aseptically, and the result was presented as mean ± standard deviation using SPSS software (version 20) of statistical analysis.

### 2.7. In Vitro Antioxidant Activity of Extracts and Isolated Compounds

In this study, extracts and isolated compounds were also subjected to antioxidative evaluation using two assays, DPPH free radical and ferric reducing antioxidant power (FRAP).

#### 2.7.1. DPPH Assaying Technique

Free radical trapping ability of extracts and compounds including a standard antioxidant agent (ascorbic acid) was checked against DPPH radical using the procedure described by Khorasani et al. [13] with some modifications. Six different concentrations, 500, 250, 150, 100, 50, and 25 *μ*g/mL, of each extract and compound isolate were prepared from corresponding stock solutions (1 mg/mL in MeOH). Same concentrations of ascorbic acid (AA) were also prepared which served as the standard antioxidant agent. To each of the above concentrations, freshly prepared DPPH solution (2 mL, 0.004% w/v in MeOH) was added followed by incubating for 30 min at room temperature. After incubation, absorbance of each concentration was measured at 517 nm using the UV-vis spectrophotometer (Cecil CE4001 UV/VIS, Cambridge, England). Sample free DPPH solution in methanol was used as negative control. The anti-DPPH free radical potential of each tested analyte was expressed in terms of percentage scavenging activity using the following formula:

(1) $$\begin{array}{c} \text{DPPH scavenging activity }\left(\text{\%}\right)=\left(1-\frac{A}{A_{0}}\right)\times 100\%, \end{array}$$

where *A* and *A*_0_ are the absorbance of DPPH with tested samples including AA and negative control (DPPH solution, 0.004% w/v in MeOH), respectively.

DPPH free radical trapping power of extracts and compounds was compared with that of ascorbic acid (AA), also expressed using IC_50_ (the concentration needed to scavenge the total DPPH radicals by 50%). Each IC_50_ value was generated from the regression equation derived from percentage scavenging activity versus concentrations graph of each tested sample. The experiment was conducted in duplicate, and results were expressed as mean ± standard deviation using the statistical analysis software of SPSS (version 20).

#### 2.7.2. Ferric Reducing Antioxidant Power (FRAP) Assay

The working principle of ferric reducing antioxidant power assay is expressed in terms of the ferric ion (Fe^3+^)-ligand complex reduction by antioxidants to the ferrous ion (Fe^2+^) complex under acidic condition, which can be monitored via the formation of intense blue color. Herein, FRAP of extracts and isolated compounds was studied using the experimental procedure applied by Do et al. [14] with some modifications. The assay involved the following experimental steps. Similar to DPPH assay mentioned above, six different concentrations (500, 250, 150, 100, 50, and 25 *μ*g/mL in H_2_O) of each sample was prepared in test tubes from stock solution (1 mg/mL in H_2_O, each). Then, each concentration was mixed with 0.2 M potassium phosphate buffer (2 mL, pH 6.6) and potassium ferricyanide (2.5 mL, 10% w/v) solutions followed by incubation at 40°C for 30 min. Trichloroacetic acid (2.5 mL, 10% w/v) was then added to the incubated solutions, centrifuged for 10 min at 3000 rpm, and a supernatant (5 mL) of each solution was mixed with distilled water (2 mL) and ferric chloride (0.5 mL, 0.1% w/v). Same preparation was also followed for the positive control, ascorbic acid. Last, absorbance of each reacted mixtures was read at 700 nm of the UV-visible spectrophotometer (Cecil CE4001 UV/VIS, Cambridge, England). The ferric ion reducing power of each tested extracts and compounds was determined by the increase in absorbance recorded in duplicate and compared with ascorbic acid. Recorded individual absorbance was subjected to SPSS (version 20) of statistical software to express as mean ± standard deviation.

### 2.8. Molecular Docking and Drug Likeness Prediction of Compounds **1** and **2**

Isolated compounds **1** and **2** were docked using the DNA gyraseB of *E. coli* as receptor cavity. The crystal structure of *E. coli* gyraseB (PDB ID: 6F86), obtained from the protein data bank (PDB), was chosen as the protein model. The molecular docking analysis was performed using the AutoDock tools (version 4.2) to predict the potential binding mode of isolated compounds into *E. coli* DNA GyrB. Polar hydrogen atoms and Gasteiger partial atomic charges were added. All rotatable bonds of the compounds, defined by default of the program, were allowed to rotate during the automated docking process. Then, prepared protein and compound structures were saved in the PDBQT format suitable for calculating energy grid maps and analyzed using AutoDock Vina. The maintaining space of the grid box was considered at 0.375 Å with the grid box size of 46 × 46 × 46  points. The Lamarckian genetic algorithm (LGA) program with an adaptive whole method search in the AutoDock was chosen to calculate the different conformers of compounds. After 200 independent docking runs for each compound, a cluster analysis was done. In accordance to the root mean squared deviation (RMSD), tolerance of 2.0 Å conformations was clustered and ranked by energy. The conformation with the best scored pose between GyrB and compounds with the lowest binding energy was considered for the compounds. The molecular docking results were analyzed based on the binding energy (kcal/mol) and number of binding interactions between catalytic amino residues and isolated compounds.

## 3. Results and Discussion

In the present investigation, two compounds **1** and **2** were isolated from methanol extract of *C. purpurea* roots, and their structures were characterized using spectroscopic techniques. The antibacterial and antioxidant activities of mentioned compounds, including extracts, were also evaluated. Additionally, molecular docking analysis of the compounds was conducted against *E. coli* gyraseB enzyme.

### 3.1. Characterization of Structures

Compound **1**: compound **1** was obtained as yellow brown amorphous (45 mg) after eluted with 100% EtOAc from MeOH roots extract of *C. purpurea*. Its TLC profile showed spot at *R*_*f*_ 0.6 using DCM/EtOAc/AcOH (1 : 1:0.1) as mobile phase. IR *ν*_max_^KBr^ cm^−1^: 1727 (ester C=O), 1282 (ester C-O), and 745 (-CH stretching of aromatic); the ^1^H, ^13^C, and DEPT-135 NMR spectral values are given in Table 1; GC-MS analysis peak at retention time of 37.03 min was observed and in the EI MS *m*/*z* (relative abundance), a molecular ion peak [M]^+^ at *m/z* of 390 was showed which is compatible with the molecular composition of C_24_H_38_O_4_, 149.0 (100%), 167 (30.31%), 57.1 (16.65%), 71.1 (11.57%), and 279.1 (10.71%).

**Figure 2:**
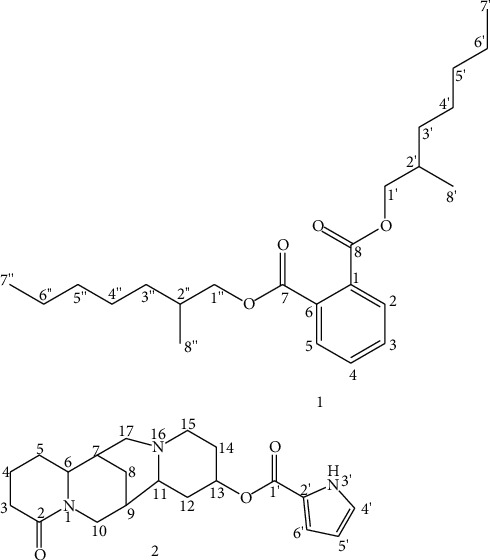
Structures of isolated compounds **1** and **2**.

In the obtained ^1^H NMR spectrum, a methylene proton signal at *δ*H 4.22–4.24 (H-1′/1″, dd, *J* = 1.82, 2.11 Hz), integrated to four protons, was observed. Besides, proton signals at *δ*H 0.96–0.98 (d, *J* = 7.28 Hz) and 0.91–0.94 (t, *J* = 10.98 Hz) integrated each to six protons were shown implying that there were four methyl groups, in which the first two (C-8′/8″-CH_3_) were attached to sp^3^ methine carbons (C-2′/2″), while the latter two (C-7′/7″-CH_3_) were found to be terminals affixed to sp^3^ methylene carbons (C-6′/6″). The presence of two symmetric sp^3^ methine groups was also confirmed by the proton signal at *δ*H 1.58–1.71 (H-2′/2″, m) in the ^1^H NMR spectrum. Other eight symmetric methylene protons were appeared at *δ*H 1.30–1.36 as multiplet integrated to a total of sixteen protons. Furthermore, two proton signals (integrated to two protons each) in the low field region at *δ*H 7.72–7.74 (H-2/5, dd, *J* = 3.68, 3.68 Hz) and 7.62–7.64 (H-3/4, dd, *J* = 3.68, 3.68 Hz) were indicative of four symmetric aryl protons having the same coupling pattern. From ^13^C and DEPT-135 NMR spectra, twelve intense carbon peaks were clearly shown, which ascribed to twenty-four carbon numbers indicating that there is a symmetry in compound **1**. For instance, the presence of a quaternary carbon signal at *δ* 167.9 (C-7/8) and methylene carbon signal at *δ* 67.7 (C-1′/1″) confirmed the occurrence of two symmetric aryl ester carbonyl groups. The ^13^C and DEPT-135 spectra also further supported this by showing eight symmetric carbons, i.e., six aromatic carbons at *δ* 132.2 (C-1/6, quaternary), 128.5 (C-2/5), and 131.0 (C-3/4). In addition, the occurrence of two carbon signals in the far right side of the ^13^C spectrum at *δ* 10.1 and 13.1 indicated that there were four equivalence methyl groups, C-8′/8″-CH_3_ (attached to C-2/'2″ at *δ* 38.8), and terminal C-7′/7″-CH_3_ (affixed to C-6′/6″ at *δ* 22.7). Moreover, other four symmetric methylene carbon peaks were observed at *δ* 23.6 (C-3′/3″), 28.7 (C-4′/4″), 30.2 (C-5′/5″), and 22.7 (C-6′/6″). In the resulted IR spectrum, the absence of a sharp intense hydroxyl peak at its absorption region and the presence of a peak at the carbonyl functional group region (1727 cm^−1^) along with the absorption bands of 1282 and 745 cm^−1^, which were due to the ester C=O, C-O and -CH stretches of aromatic protons, respectively, directed that compound **1** exhibits a diester of phthalic acid. Based on the above discussed spectral data (Table 1), the structure of compound **1** was deduced as di-(2-methylheptyl) phthalate (Figure 2), which was also reported by Rameshthangam and Ramasamy [15] in which the spectroscopic data were found in a good agreement with compound **1**. Eventhough di-(2-methylheptyl) phthalate is a member of the phthalate groups, unlike to other phthalate esters, it is not a petrochemical and plasticizer. Most of the phthalates and their derivatives are petrochemicals which have been used as plasticizers in chemical industry to improve the plasticity and flexibility of the industrial products [16, 17]. For the past several decades, phthalates have been known as totally synthetic compounds and environmental pollutants of industrial origin than biogenic sources. However, this perception has been changed following the isolation of many phthalic compounds like di-(2-ethylhexyl) phthalate [18] and dibutyl phthalate (DBP) [17], from unpolluted natural sources such as algae, bacteria, fungi, and higher plants. Owing to this, there are controversies among researchers on the real origin of these compounds. Some argue that those phthalates isolated from living organisms are industry origins which leached to the environment and later absorbed by the organisms. Others have different points of view saying that phthalates can be biogenic or endogenous naturally originated from the biosources [17, 19]. The latter idea has been supported by a reported experimental evidence in which natural phthalate biosynthesis was observed in two labeled alga via the analysis of the natural abundance ^14^C content of two isolated phthalates, dibutyl phthalate (DBP) and di-(2-ethylhexyl) phthalate (DEHP), and compared with industry originated standard plasticizers of the two. The shikimic acid pathway was the proposed and recognized biosynthetic pathway of the phthalates like DBP [17, 20]. However, researchers still believe that further experimental evidences needed to judge phthalates can really be natural products than they are totally anthropogenic origin.

Compound **2**: compound **2** eluted with a 1 : 1 ratio of EtOAc/MeOH from MeOH roots extract yielded a yellow powder (300 mg); m.p. 151–154^o^ ([21], 153–154^o^); *R*_f_ value: 0.2 (EtOAc/MeOH/AcOH; 1 : 1:0.1); IR *ν*_max_^KBr^ cm^−1^: 3342 (-NH), 2918–2855 (-CH stretching of trans-quinolizidine), 1692 (ester C=O), and 1616 (lactam C=O). Detailed ^1^H, ^13^C, and DEPT-135 spectral data are given in Table 2; GC-MS analysis peak at retention time of 38.66 min was observed, and in the EI MS *m*/*z* (relative abundance), [M]^+^ (357.3, C_20_H_27_N_3_O_3_) was observed, 246.2 (100%), 134.05 (29.86%), 112.0 (weak), and 94.0 (weak).

In the ^1^H NMR spectrum, a triplet of triplet methylene proton signal was observed in the relatively downfield shift at *δ*H 4.47–4.52 (H-10, tt, *J* = 4.46, and 4.46 Hz) along with a methylene carbon signal at the aliphatic region (*δ* 46.6, C-10) in the ^13^C spectrum. Besides, the relatively deshielded multiplet (*δ*H 3.63–3.69, H-6) and triplet (*δ*H 5.19–5.21, H-13, *J* = 5.39) proton signals indicate the presence of methine groups attached to heteroatoms (-CH-N- and -CH-O-, respectively), as also reported elsewhere [22]. Furthermore, eight additional methylene and three methine protons were also observed as multiplets in the aliphatic region (*δ*H 1–3 ppm). From the ^13^C and DEPT-135 spectra, fifteen clear carbon signals were shown which represent a quaternary carbonyl carbon of the amide group at *δ* 172.8 (C-2), nine sp^3^ methylene carbons at *δ* 32.3 (C-3), 18.9 (C-4), 25.6 (C-5), 26.7 (C-8), 46.6 (C-10), 35.4 (C-12), 49.4 (C-15), 27.7 (C-14), and 51.2 (C-17), and five sp^3^ methine carbons appeared at *δ* 60.7 (C-6), 33.9 (C-7), 32.2 (C-9), 57.9 (C-11), and 67.7 (C-13). Generally, the abovementioned proton and carbon signals were complementary and characteristics of lupanine skeleton. Besides, the absorption bands shown at 1616 and 2918–2855 cm^−1^ in the IR spectrum, which belong to the amide carbonyl group (lactam C=O) and -CH stretch of the trans-quinolizidine, respectively, highlighted that compound **2** exhibits a lupanine skeleton. As also reported elsewhere [22], an ester of pyrrolecarboxylic acid was identified in compound **2** which was assured by three doublets of doublet proton signals occurred at *δ*H 7.01 (H-4′, dd, *J* = 1.58, 1.37 Hz), 6.21–6.23 (H-5′, dd, *J* = 2.26, 2.26 Hz), and 6.90–6.92 (H-6′, dd, *J* = 1.32, 1.64 Hz) assigned to the three olefinic methine protons of the pyrrolecarboxyl unit. Their corresponding carbon peaks appeared at *δ* 123.5 (C-4′), 109.4 (C-5′) and 115.6 (C-6′). In the DEPT-135 spectrum, the two quaternary carbon signals shown at *δ* 122.2 and 161.7 belong to C-2′ and the carbonyl carbon (C-1′), respectively, of the substituent. Also, the carbonyl (C=O) and secondary -NH groups of the esterified pyrrolecarboxyl moiety showed absorption bands in the wavenumbers (cm^−1^) of 1692 and 3342 (broad), respectively, in the IR spectrum. This esterified pyrrolecarboxyl unit was incorporated to the lupanine skeleton of compound **2** at C-13 which was identified by the relatively deshielded carbon signal at *δ* 67.7 (C-13) and triplet proton signal of H-13 at *δ*H 5.19–5.21 (*t*, *J* = 5.39 Hz). The detailed spectral information obtained of compound **2** is given in Table 2. With the help of the present spectral data and reported information [22], the structure of compound **2** was characterized as 13-O-pyrrolecarboxyl lupanine (Figure 2). The spectral data and structure of this compound were found in a good match with calpurnine compound reported by Nasution and Kinghorn [22].

### 3.2. Biological Activities of Extracts and Isolated Compounds

#### 3.2.1. Antibacterial Activity

The antibacterial activity of the extracts and isolated compounds was evaluated following the Mueller–Hinton agar disc diffusion method. The results are given in Tables 3 and 4. In principle, extracts/isolated compounds showing an inhibitory diameter zone of ≥7 mm are considered active against tested bacterial strains [23]. Herein also, extracts and compounds exhibiting mean of inhibition zones with diameter of greater than 7 mm were taken as active on the evaluated bacteria at a given concentration(s).

Table 3 provides that *n*-hexane, chloroform, and chloroform/methanol (1 : 1) extracts were found effective against *E. coli* (>7 mm inhibition zone) at all tested concentrations with the maximum inhibition diameters of 13.8 ± 0.0 mm, 11.1 ± 0.0 mm, and 10.7 ± 0.1 mm, respectively, recorded at the maximal concentration of 100 mg/mL, which were slightly comparable to chloramphenicol (24.5 ± 0.3 mm at 30 *μ*g dose). The remaining lower concentrations, 50 mg/mL, 25 mg/mL, and 12.5 mg/mL, of these extracts also produced an activity with corresponding inhibition zone values of 10.2 ± 0.4 mm, 9.3 ± 0.0 mm, and 8.7 ± 0.0 mm recorded by *n*-hexane extract, 9.6 ± 0.3 mm, 8.3 ± 0.0 mm, and 7.2 ± 0.0 mm recorded by chloroform extract, and 9.0 ± 0.3 mm, 7.2 ± 0.0 mm, and 7.0 ± 0.0 mm recorded by chloroform/methanol (1 : 1) extract. Interestingly, both the alcoholic extracts experienced a lesser activity against *E. coli* at concentrations of 100 mg/mL and 50 mg/mL with respective inhibitory values of 9.6 ± 0.1 mm and 8.3 ± 0.1 mm for the methanol extract and 8.0 ± 0.0 mm and 7.6 ± 0.0 mm for the ethanol extract, while no inhibited area was visible at the remaining concentrations of the extracts. The Gram-positive *S. aureus* strain was found to be susceptible only to the *n*-hexane and methanol extracts up to the 25 mg/mL dose with measured inhibited area ranging from 7.6 ± 0.0 mm to 10.2 ± 0.5 mm and 7.3 ± 0.0 mm to 8.7 ± 0.0 mm, respectively. Whereas, completely no cleared zone was observed at the minimal concentration (12.5 mg/mL) of these extracts. Also, all tested concentrations of chloroform, chloroform/methanol (1 : 1), and ethanol extracts showed a totally zero zone of inhibition against *S. aureus*. On the other hand, the chloroform/methanol (1 : 1) extract exerted a positive action on the growth of *P. aeruginosa* bacterium at all tested dilutions with measured cleared area of 10.0 ± 0.1 mm at 100 mg/mL, 9.7 ± 0.1 mm at 50 mg/mL, 8.4 ± 0.2 mm at 25 mg/mL, and 8.0 ± 0.0 mm at 12.5 mg/mL. The chloroform and *n*-hexane extracts also made an attempt to inhibit some area of *P. aeruginosa* growth at all concentrations by providing measured values of 7.7 ± 0.0 mm–9.8 ± 0.1 mm and 7.3 ± 0.0 mm–8.9 ± 0.2 mm, respectively. However, the methanolic and ethanolic extracts scored a slightly lower inhibitory values (7.4 ± 0.0 mm–8.7 ± 0.1 mm and 7.1 ± 0.0 mm–8.3 ± 0.1 mm, respectively) against *P. aeruginosa* at the doses up to 25 mg/mL, but a nil inhibition zone at the least concentration (12.5 mg/mL). To summarize, a better activity against *E. coli*, especially at the higher concentrations (100 mg/mL and 50 mg/mL), was noted in the nonalcoholic extracts though it looked like less as compared to chloramphenicol (24.5 ± 0.3 mm at dose of 30 *μ*g). Whereas, the inhibitory action indicated by almost all extracts against *S. aureus* at all concentrations was noted weak in reference to chloramphenicol (18.8 ± 0.4 mm). To the contrary, the inhibition zone values, scored by the extracts against *P. aeruginosa*, were found even better than that of chloramphenicol (7.2 ± 0.6 mm).

As given in Table 4, compound **2** presented better inhibitory activity than compound **1** against *E. coli* with greater zone of inhibition of 18.5 ± 0.0 mm recorded at the higher concentration of 1.0 mg/mL, which was comparable to chloramphenicol (21.5 ± 0.3 mm at dose of 30 *μ*g). The remaining concentrations, 0.5 mg/mL, 0.3 mg/mL, 0.1 mg/mL, and 0.05 mg/mL, of compound **2** also produced an activity against same bacterium with respective mean inhibition values of 16.2 ± 0.0 mm, 13.8 ± 0.0 mm, 10.0 ± 0.0 mm, and 9.6 ± 0.0 mm. Compound **1** displayed a moderate activity against *E. coli* at all concentrations (>7 mm) with inhibition zones laid in the range of 7.2 ± 0.1 mm–10.3 ± 0.0 mm. Whereas, against *S. aureus*, compound **1** showed a minor inhibitory effect only at concentrations of 1.0 mg/mL (9.1 ± 0.1 mm) and 0.5 mg/mL (8.4 ± 0.0 mm), but a zero effect at the remaining concentrations. Compound **2** was found totally inactive against *S. aureus* at all concentrations with nil inhibition zone values. However, the two compounds possessed an activity stronger than the chloramphenicol (7.2 ± 0.6 mm) against *P. aeruginosa* bacterium almost at all dilutions, that is, compound **2** produced mean inhibition zone values ranging from 7.2 ± 0.6 mm to 10.6 ± 0.0 mm and that of compound **1** was found between 7.1 ± 0.0 mm and 9.0 ± 0.1 mm. Table 4, in general, revealed that compound **2** provided a notable activity against *E. coli*, especially at the higher concentration, which resulted in an inhibition value close to that of chloramphenicol; whereas, compound **1** was found with lesser activity as compared with compound **2** and chloramphenicol. However, against the methicillin resistant *S. aureus* strain, both compounds exhibited a negligible activity up to 1.0 mg/mL in comparison to the chloramphenicol antibiotic (18.8 ± 0.4 mm at the dose of 30 *μ*g). But, surprisingly, chloramphenicol had faced strong and unprecedented resistant from *P. aeruginosa* bacterium which led to even a smaller inhibition zone value (7.2 ± 0.6 mm) than that of the compounds. Overall, the resulted inhibitory potential of the extracts and isolated compounds examined herein was found slightly comparable against *E. coli*, very weak against *S. aureus*, and stronger against *P. aeruginosa* in reference to the standard antibiotic drug, chloramphenicol (at 30 *μ*g dose). In fact, it is difficult to exactly compare the antimicrobial potency of extracts and/or isolates directly with synthesized standard antibiotics like chloramphenicol for similar doses, since there are huge differences between them in terms of chemical polarity, structural patterns, and others.

#### 3.2.2. Antioxidant Activity

In vitro antioxidant potential of four extracts (chloroform, 1 : 1 of chloroform/methanol, methanol, and ethanol) and two isolated compounds **1** and **2** of *Cadia purpurea* roots was evaluated against DPPH free radical and ferric ion oxidation. Six various dilutions (500, 250, 150, 100, 50, and 25 *μ*g/mL) of each extract and compound, including ascorbic acid, were prepared from their respective stock solutions (1 mg/mL). Each solution was subjected to the UV-Vis spectrophotometer to measure their absorbance against DPPH radical and ferric reducing power at 517 and 700 nm, respectively. Resulted values of duplicates were expressed as mean ± standard deviation.

**Figure 3:**
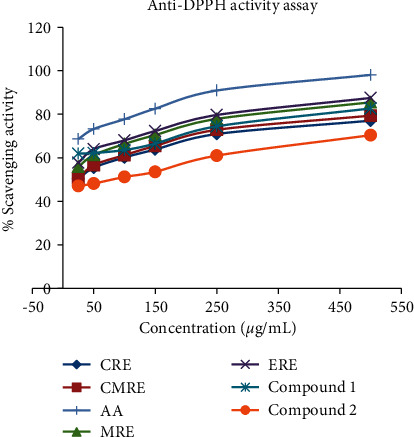
DPPH radical scavenging activity percentage (mean of duplicates) against various concentrations of *Cadia purpurea* roots extracts and isolated compounds. CRE, CMRE, MRE, and ERE, respectively, are chloroform, chloroform/methanol (1 : 1), methanol, and ethanol roots extracts, and AA is the ascorbic acid standard.

DPPH free radical scavenging activity: the DPPH free radical scavenging potential of extracts and isolated compounds was determined compared to ascorbic acid (AA). The obtained result showed that activity of the extracts and compounds against DPPH radical was dose-dependent, in which the DPPH scavenging activity percentage was directly correlated with concentrations (Table 5 and Figure 3).

As given in Table 5 and Figure 3, the alcoholic extracts (ethanol and methanol) exhibited a better trapping activity percentage (87.51 ± 0.11 and 85.51 ± 0.11, respectively) on the DPPH radical with respective IC_50_ values of 12.9 and 16.03 *μ*g/mL at 500 *μ*g/mL concentration. The chloroform and chloroform/methanol (1 : 1) extracts provided the comparatively lesser anti-DPPH activity percentage of 77.07 ± 0.00 (IC_50_ value, 27.52 *μ*g/mL) and of 79.38 ± 0.16 (26.14 *μ*g/mL of IC_50_) at same concentration. In reference to the observed anti-DPPH potency of ascorbic acid (98.10 ± 0.00, IC_50_ value of 4.82 *μ*g/mL) at 500 *μ*g/mL, however, the inhibitory activity against the free radical of the tested extracts was found to be weak.

Of the compounds evaluated, compound **1** presented a greater anti-DPPH activity percentage of 82.69 ± 0.11 at concentration of 500 *μ*g/mL with an IC_50_ value of 7.99 *μ*g/mL. The quinolizidine alkaloid compound **2** exhibited the smaller scavenging percentage value (70.40 ± 0.00, IC_50_ of 58.34 *μ*g/mL) (Table 5 and Figure 3). Here, the smaller IC_50_ value implies the better DPPH radical scavenging activity and vice versa. Hence, the overall antioxidative activity against the DPPH radical of the compounds was observed much weaker as compared to the ascorbic acid standard (IC_50_ value of 4.82 *μ*g/mL) for same concentration.

Ferric reducing antioxidant power examination: the potential of reducing ferric (Fe^3+^) ion into its ferrous (Fe^2+^) of extracts and isolates was known by first observing a change in color of the reaction solutions from yellow to green and then measuring the absorbance at 700 nm [24]. The ferric reducing antioxidant power, in terms of the mean absorbance value, of tested extracts and isolated compounds is given in Table 6. The recorded reduction absorbance against ferric ion of each tested sample was obtained positively related with corresponding concentrations, that is, the increase in sample concentration led to the intense green color, and high absorbance value of solution suggests a strong ferric ion reducing potential. Alike to DPPH radical scavenging percentage, the alcoholic extracts exhibited stronger ferric ion reducing antioxidant activity with the higher absorbance values of 0.810 ± 0.001 and 0.788 ± 0.000 observed in the methanol and ethanol extracts, respectively, at the higher concentration (Table 6). With respect to the standard ascorbic acid (2.225 ± 0.000), however, observed ferric reduction power of studied extracts was not remarkable.

Compound **1** indicated a notable ferric ion reduction potential with an absorbance value of 0.761 ± 0.002 at the concentration of 500 *μ*g/mL. Whereas, compound **2** showed a comparatively weak reduction strength (0.458 ± 0.001) at same amount (Table 6). However, the overall ferric reducing antioxidant power (in terms of absorbance) of the compounds was found to be weak compared with that of ascorbic acid at similar concentrations.

### 3.3. Molecular Docking Study

**Figure 4:**
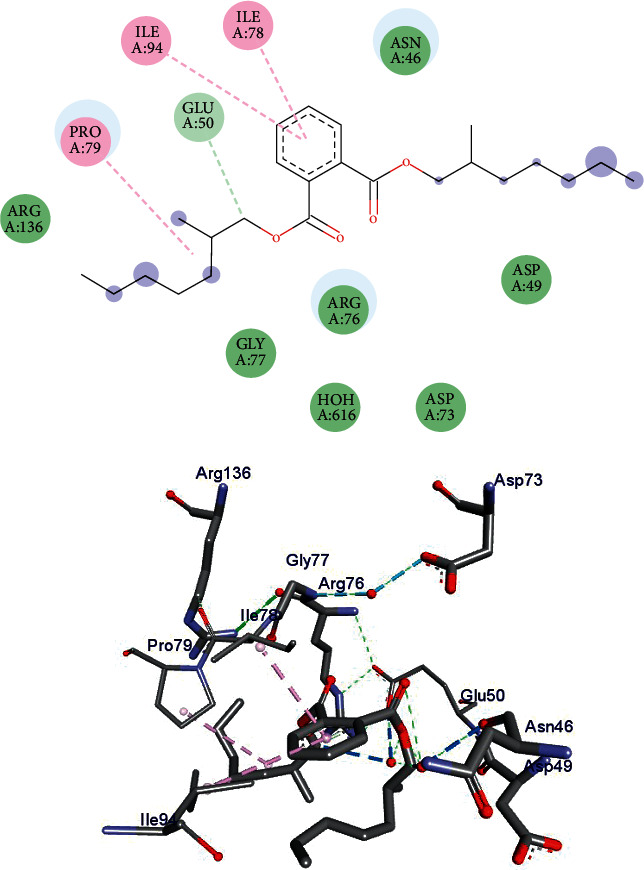
Ligand interaction (top) and 3D representation (bottom) of compound **1** into *E. coli* DNA gyraseB cleavage complex.

**Figure 5:**
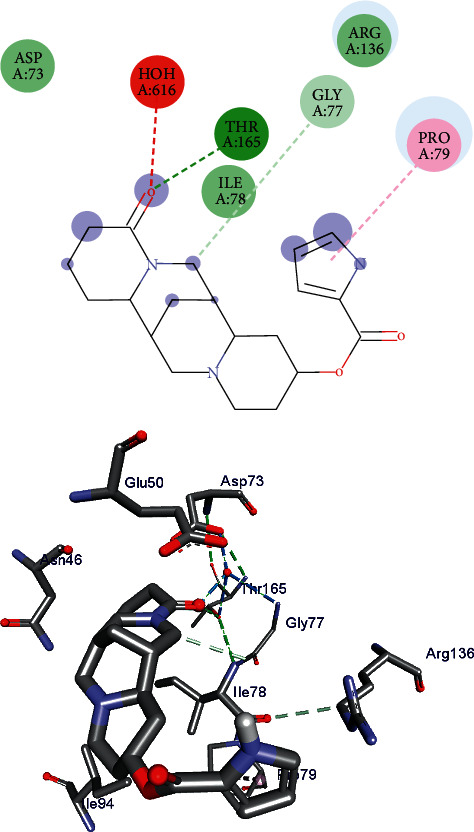
Ligand interaction (top) and 3D representation (bottom) of compound **2** into DNA gyraseB protein of *E. coli*.

Isolated compounds **1** and **2** were subjected to molecular docking analysis to predict their drug-likeness properties (based on binding energy and binding interactions) toward DNA gyraseB protein of *E. coli* selected from the Protein Data Bank (PDB ID: 6F86). The docking analysis result (Figure 4) revealed that compound **1** shows two hydrophobic interactions with Ile-78 and Ile-94 and a *π*-anion with Glu-50 amino residues of the GyrB enzyme. A coordination between Pro-79 and compound **1** is also observed. In addition, compound **1** binds to 6F86 amino residues Asp-73, Asp-49, Arg-76, Gly-77, Asn-46, and Arg-36 via Van der Walls interaction. The binding affinity towards *E. coli* DNA GyrB of compound **1** is found to be −5.4 kcal/mole. Whereas, a heavy negative binding energy (−7.4 kcal/mole) is observed in compound **2** which is comparable with that of ciprofloxacin (−7.2 kcal/mole). As shown in Figure 5, compound **2** makes three H-bond interactions with Thr-165 via water, Gly-77, and Ile-78 residual amino acids. Besides, this compound coordinates to Pro-79 including a Van der Walls interaction with Asp-73. The hydrophobic and *π*-anion binding ability of compound **1** with Ile-78 and Glu-50 binding pockets, respectively, is the only drug-likeness indicator of the compound in comparison to ciprofloxacin. Whereas, the ciprofloxacin-like property of compound **2** is recognized only via the formation of a H-bond with Thr-165 active binding site of 6F86.

## 4. Conclusion

In this study, two compounds, di-(2-methylheptyl) phthalate (**1**) and 13-O-pyrrolecarboxyl lupanine (**2**), were isolated from methanolic extract of *Cadia purpurea* roots for the first time. In vitro antibacterial and antioxidant activities of isolated compounds and crude extracts were also evaluated, and the results showed that *n*-hexane, chloroform, and chloroform/methanol (1 : 1) extracts were found effective against *E. coli* at all tested concentrations with the maximum inhibition diameters of 13.8 ± 0.0 mm, 11.1 ± 0.0 mm, and 10.7 ± 0.1 mm, respectively, recorded at the maximal concentration of 100 mg/mL, which were slightly comparable to chloramphenicol (24.5 ± 0.3 mm at 30 *μ*g dose). The methanolic and ethanolic extracts, however, experienced a smaller activity against *E. coli* merely at concentrations of 100 mg/mL and 50 mg/mL with respective inhibitory values of 9.6 ± 0.1 mm and 8.3 ± 0.1 mm, and 8.0 ± 0.0 mm and 7.6 ± 0.0 mm. The methicillin resistant *S. aureus* strain was found to be sensitive only to the *n*-hexane and methanol extracts up to the 25 mg/mL dose with measured inhibited area ranging from 7.6 ± 0.0 mm to 10.2 ± 0.5 mm, and 7.3 ± 0.0 mm to 8.7 ± 0.0 mm, respectively. Whereas the chloroform, chloroform/methanol (1 : 1), and ethanol extracts did not indicate any sign of activity against *S. aureus* even at the higher concentration of 100 mg/mL. On the other hand, the chloroform/methanol (1 : 1) extract exerted an inhibitory action on the growth of *P. aeruginosa* bacterium at all tested dilutions with measured clear area of 8.0 ± 0.0 mm–10.0 ± 0.1 mm. The chloroform and *n*-hexane extracts also attempted to inhibit some growth of *P. aeruginosa* at all concentrations with corresponding measured values of 7.7 ± 0.0 mm–9.8 ± 0.1 mm and 7.3 ± 0.0 mm–8.9 ± 0.2 mm. However, the methanolic and ethanolic extracts scored a slightly lower inhibitory values (7.4 ± 0.0 mm–8.7 ± 0.1 mm and 7.1 ± 0.0 mm–8.3 ± 0.1 mm, respectively) against *P. aeruginosa* up to 25 mg/mL concentration. Compound **2** presented a better inhibitory effect against *E. coli* than compound **1** with a comparable inhibition value (18.5 ± 0.0 mm) to chloramphenicol (21.5 ± 0.3 mm at dose of 30 *μ*g) recorded at the higher concentration of 1.0 mg/mL. This compound **2** also produced an activity on same bacterium at the concentrations of 0.5 mg/mL, 0.3 mg/mL, 0.1 mg/mL, and 0.05 mg/mL with zone of inhibition values ranging between 9.6 ± 0.0 mm and 16.2 ± 0.0 mm. Compound **1** displayed a moderate activity (>7 mm) against *E. coli* at all concentrations with inhibition zones laid in the range of 7.2 ± 0.1 mm–10.3 ± 0.0 mm. Whereas, against *S. aureus*, compound **1** showed a minor inhibitory effect only at concentrations of 1.0 mg/mL (9.1 ± 0.1 mm) and 0.5 mg/mL (8.4 ± 0.0 mm) and compound **2** was found totally inactive at all concentrations. The two compounds, however, possessed an activity stronger than the chloramphenicol (7.2 ± 0.6 mm) against *P. aeruginosa* bacterium almost at all dilutions with mean inhibition zone values ranging from 7.2 ± 0.6 mm to 10.6 ± 0.0 mm of compound **2** and 7.1 ± 0.0 mm to 9.0 ± 0.1 mm of compound **1**. The obtained antibacterial result generally revealed that nonalcoholic extracts were noted with a better activity against *E. coli*, especially at the higher concentrations (100 mg/mL and 50 mg/mL), though they seemed like weak compared to the chloramphenicol (24.5 ± 0.3 mm at dose of 30 *μ*g). Compound **2** also provided a notable activity against *E. coli*, especially at the higher concentration, which resulted in an inhibition value close to that of chloramphenicol (21.5 ± 0.3 mm); whereas, compound **1** was found with weaker activity as compared with compound **2** and chloramphenicol. On the other hand, against the methicillin resistant *S. aureus* strain, both compounds and all the extracts exhibited a negligible activity up to the higher dosage in reference to the chloramphenicol antibiotic (18.8 ± 0.4 mm). On the contrary, the inhibition zone values, scored by the extracts and compounds against *P. aeruginosa*, were found even better than that of chloramphenicol (7.2 ± 0.6 mm). Regarding the antioxidant potency, ethanolic and methanolic extracts exhibited a better DPPH radical scavenging activity (IC_50_ values of 12.9 and 16.03 *μ*g/mL, respectively) at concentration of 500 *μ*g/mL, whereas the chloroform and chloroform/methanol (1 : 1) extracts were observed weak with IC_50_ values of 27.52 and 26.14 *μ*g/mL. Comparatively strong ferric ion reducing power was also shown in the alcoholic extracts with 0.810 ± 0.001 absorbance of methanol and 0.788 ± 0.000 of ethanol at the maximum amount (500 *μ*g/mL). Compound **1** indicated a promising trapping potential against DPPH radical (IC_50_ value, 7.99 *μ*g/mL), while compound **2** recorded a higher IC_50_ value (58.34 *μ*g/mL) at concentration of 500 *μ*g/mL. However, at same dosage, the isolated compounds indicated a weak power of ferric ion reduction with recorded absorbance of 0.761 ± 0.002 by compound **1** and 0.458 ± 0.001 of compound **2**. The molecular docking result revealed that compound **1** shows two hydrophobic (with Ile-78 and Ile-94), a *π*-anion (with Glu-50) and Van der Walls interactions with some amino residues of *E. coli* gyraseB; whereas, compound **2** makes three H-bond interactions with Thr-165, Gly-77, and Ile-78 in addition to the Van der Walls interaction with Asp-73. In conclusion, the present findings indicated that the resulted antibacterial inhibitory potential of the extracts and isolated compounds was found slightly active against *E. coli*, very weak against *S. aureus*, and stronger against *P. aeruginosa* as compared to chloramphenicol (at 30 *μ*g dose); and the entire antibacterial and antioxidant activities of tested analytes were noted as dose-dependent. Besides, since other quinolizidine alkaloids (similar to compound **2**) were also reported from the leaves of Ethiopian *Cadia purpurea*, conducting further biochemical investigations on all parts of the plant could provide opportunities of finding additional alkaloidal compounds and other phthalates with powerful biological activities.

## Figures and Tables

**Table 1:** ^1^H (400 MHz), ^13^C, and DEPT-135 (100 MHz) NMR data (in CD_3_OD, *δ* in ppm, *J* in Hz) of compound (**1**).

Compound (**1**)				Literature information [15]	
Attribution	*δ* ^1^H (multiplicity and coupling)	*δ* ^13^C	DEPT-135	*δ* ^1^H	*δ* ^13^C
1′/1″	4.22–4.24 (dd, *J* = 1.82, 2.11)	67.7	-CH_2_-O-	4.15 (m)	68.1
2′/2″	1.58–1.71 (m)	38.8	-CH-	1.25 (m)	38.7
3′/3″	1.30–1.36 (m)	23.6	-CH_2_-	1.25 (m)	23.7
4′/4″	1.30–1.36 (m)	28.7	-CH_2_-	1.25 (m)	28.9
5′/5″	1.30–1.36 (m)	30.2	-CH_2_-	1.25 (m)	30.3
6′/6″	1.30–1.36 (m)	22.7	-CH_2_-	1.25 (m)	22.9
7′/7″	0.91–0.94 (t, *J* = 10.98)	13.1	-CH_3_	0.82	14.1
8′/8″	0.96–0.98 (d, *J* = 7.28)	10.1	-CH_3_	0.82	10.9
7/8	—	167.9	Q (C=O)	—	167.8
1/6	—	132.2	Q	—	130.9
2/5	7.72–7.74 (dd, *J* = 3.68, 3.68)	128.5	= CH-	7.65 (dd)	128.8
3/4	7.62–7.64 (dd, *J* = 3.68, 3.68)	131.0	= CH-	7.45 (dd)	132.4

**Table 2:** ^1^H (400 MHz), ^13^C, and DEPT-135 (100 MHz) NMR spectral information (in CD_3_OD, *δ* in ppm, *J* in Hz) of compound **2**.

Compound **2**				Calpurnine [22]
C/H	*δ* ^1^H	*δ* ^13^C	DEPT-135	*δ*13C
1	—	—	—	—
2	—	172.8	Q (C=O)	171.6
3	2.53–2.60 (m)	32.3	-CH_2_-C=O	33.1
4	1.62–1.71 (m)	18.9	-CH_2_-	19.5
5	1.62–1.71 (m)	25.64	-CH_2_-	26.6
6	3.63–3.69 (m)	60.7	-CH-N-	60.7
7	1.86–1.89 (m)	33.9	-CH-	34.2
8	1.42–1.49 (m)	26.7	-CH_2_-	27.3
9	1.86–1.89 (m)	32.2	-CH-	32.6
10	4.47–4.52 (tt, *J* = 4.46, 4.46)	46.6	-CH_2_-N-	46.9
11	2.65–2.69 (m)	57.9	-CH-N-	57.6
12	2.21–2.25 (m)	35.4	-CH_2_-C-O-	36.1
13	5.19–5.21 (t, *J* = 5.39)	67.7	-CH-O-	68.0
14	2.40–2.45 (m)	27.7	-CH_2_-C-O-	28.7
15	2.72–2.77 (m)	49.4	-CH_2_-	49.9
16	—	—	—	—
17	3.02–3.07 (m)	51.2	-CH_2_-N-	52.1

13-Pyrrolecarboxyl				
1′	—	161.7	Q (C=O)	160.1
2′	—	122.2	Q	122.9
3′	5.01 (br *s*, 20 amine)	—	—	—
4′	7.01 (dd, *J* = 1.58, 1.37)	123.5	= CH-NH	123.4
5′	6.21–6.23 (dd, *J* = 2.26, 2.26	109.4	= CH-	110.3
6′	6.90–6.92 (dd, *J* = 1.32, 1.64)	115.6	= CH-	116.1

**Table 3:** Antibacterial inhibitory action (mean ± SD, in mm) on standard *E. coli*, *S. aureus*, and *P. aeruginosa* of five extracts.

Bacterial culture	Concentration (mg/mL)	Diameter of inhibition area in mm (mean ± SD) of extracts and chloramphenicol					
		*n*-Hexane	Chloroform	Chloroform: methanol (1 : 1)	Methanol	Ethanol	Chloramphenicol (30 *μ*g/disc)
*E. coli*	12.5	8.3 ± 0.0	7.2 ± 0.0	7.0 ± 0.0	6.5	0.0	24.5 ± 0.3
	25	9.3 ± 0.0	8.7 ± 0.0	7.2 ± 0.0	6.8 ± 0.0	0.0	
	50	10.2 ± 0.4	9.6 ± 0.3	9.0 ± 0.3	8.3 ± 0.1	7.6 ± 0.0	
	100	13.8 ± 0.0	11.1 ± 0.4	10.7 ± 0.1	9.6 ± 0.1	8.0 ± 0.0	

*S. aureus*	12.5	0.0	0.0	0.0	0.0	0.0	18.8 ± 0.4
	25	7.6 ± 0.0	0.0	0.0	7.3 ± 0.0	0.0	
	50	8.9 ± 0.1	0.0	0.0	7.5 ± 0.0	0.0	
	100	10.2 ± 0.5	0.0	0.0	8.7 ± 0.0	0.0	

*P. aeruginosa*	12.5	7.3 ± 0.2	7.7 ± 0.0	8.0 ± 0.0	0.0	0.0	7.2 ± 0.6
	25	7.7 ± 0.0	8.2 ± 0.1	8.4 ± 0.2	7.4 ± 0.0	7.1 ± 0.0	
	50	8.4 ± 0.1	8.9 ± 0.0	9.7 ± 0.1	7.7 ± 0.1	7.4 ± 0.0	
	100	8.9 ± 0.2	9.8 ± 0.1	10.0 ± 0.1	8.7 ± 0.1	8.3 ± 0.1	

**Table 4:** Antibacterial activity inhibition zone (mean ± SD, in mm) of isolated compounds against standard *E. coli*, *S. aureus*, and *P. aeruginosa* bacterial strains.

Bacterial pathogens	Concentration (mg/mL)	Inhibition zone (mean ± SD, in mm) of compounds		
		Compound (**1**)	Compound (**2**)	Chloramphenicol disc (30 *μ*g/disc)
*E. coli*	0.05	7.2 ± 0.1	9.6 ± 0.0	21.5 ± 0.3
	0.1	7.5 ± 0.0	10.0 ± 0.0	
	0.3	8.6 ± 0.0	13.8 ± 0.0	
	0.5	8.9 ± 0.0	16.2 ± 0.0	
	1.0	10.3 ± 0.0	18.5 ± 0.0	

*S. aureus*	0.05	0.0	0.0	18.8 ± 0.4
	0.1	0.0	0.0	
	0.3	0.0	0.0	
	0.5	8.4 ± 0.0	0.0	
	1.0	9.1 ± 0.1	0.0	

*P. aeruginosa*	0.05	7.1 ± 0.0	7.2 ± 0.0	7.2 ± 0.6
	0.1	7.3 ± 0.0	7.5 ± 0.0	
	0.3	7.6 ± 0.0	8.6 ± 0.0	
	0.5	7.7 ± 0.0	9.1 ± 0.1	
	1.0	9.0 ± 0.1	10.6 ± 0.0	

**Table 5:** DPPH radical scavenging percentage (mean ± standard deviation) activity of extracts and isolated compounds.

Concentration (*μ*g/mL)			% scavenging activity (mean ± SD) against DPPH radical of extracts and compounds				
	CHCl_3_	CHCl_3_/MeOH (1 : 1)	Methanol	Ethanol	Compound (**1**)	Compound (**2**)	Ascorbic acid
25	50.28 ± 0.06	50.56 ± 0.11	55.94 ± 0.00	57.77 ± 0.06	61.96 ± 0.11	47.08 ± 0.11	68.66 ± 0.11
50	55.46 ± 0.00	56.67 ± 0.11	61.26 ± 0.11	63.98 ± 0.06	62.20 ± 0.06	48.17 ± 0.16	73.24 ± 0.16
100	60.16 ± 0.22	61.22 ± 0.00	66.36 ± 0.06	68.01 ± 0.11	63.59 ± 0.1	51.14 ± 0.11	77.74 ± 0.11
150	63.83 ± 9.06	65.47 ± 0.16	70.47 ± 0.06	72.33 ± 0.11	66.52 ± 0.00	53.56 ± 0.06	82.61 ± 0.00
250	70.94 ± 0.06	72.86 ± 0.11	77.89 ± 0.06	79.68 ± 0.06	74.47 ± 0.06	61.02 ± 0.06	90.92 ± 0.06
500	77.07 ± 0.00	79.38 ± 0.16	85.51 ± 0.11	87.51 ± 0.11	82.69 ± 0.11	70.4 ± 0.00	98.10 ± 0.00
IC_50_ (*μ*g/mL)	27.52	26.14	16.03	12.9	7.99	58.34	4.82

**Table 6:** FRAP (mean ± standard deviation) of *Cadia purpurea* roots extracts and isolated compounds.

Concentration (*μ*g/mL)			Absorbance (mean ± standard deviation, at 700 nm) of roots extracts and compounds				
	CHCl_3_	CHCl_3_/MeOH (1 : 1)	MeOH	EtOH	Compound (**1**)	Compound (**2**)	Ascorbic acid
25	0.308 ± 0.001	0.429 ± 0.001	0.460 ± 0.001	0.424 ± 0.001	0.497 ± 0.001	0.409 ± 0.001	0.911 ± 0.001
50	0.308 ± 0.001	0.524 ± 0.001	0.472 ± 0.001	0.448 ± 0.000	0.510 ± 0.001	0.411 ± 0.001	0.925 ± 0.001
100	0.351 ± 0.000	0.542 ± 0.002	0.506 ± 0.000	0.508 ± 0.002	0.514 ± 0.001	0.416 ± 0.000	1.069 ± 0.002
150	0.381 ± 0.001	0.573 ± 0.001	0.549 ± 0.000	0.539 ± 0.001	0.612 ± 0.001	0.422 ± 0.001	1.303 ± 0.002
250	0.381 ± 0.000	0.610 ± 0.002	0.563 ± 0.002	0.514 ± 0.001	0.623 ± 0.001	0.425 ± 0.000	1.855 ± 0.001
500	0.466 ± 0.001	0.653 ± 0.001	0.810 ± 0.001	0.788 ± 0.000	0.761 ± 0.002	0.458 ± 0.001	2.225 ± 0.000

## Data Availability

The data used to support the findings of this study are available from the corresponding author upon request.
